# Melatonin Regulates Root Meristem by Repressing Auxin Synthesis and Polar Auxin Transport in *Arabidopsis*

**DOI:** 10.3389/fpls.2016.01882

**Published:** 2016-12-15

**Authors:** Qiannan Wang, Bang An, Yunxie Wei, Russel J. Reiter, Haitao Shi, Hongli Luo, Chaozu He

**Affiliations:** ^1^Hainan Key Laboratory for Sustainable Utilization of Tropical Bioresources, College of Agriculture, Hainan UniversityHaikou, China; ^2^Department of Cell Systems and Anatomy, The University of Texas Health Science CenterSan Antonio, TX, USA

**Keywords:** melatonin, auxin, root meristem, auxin synthesis, polar auxin transport, *Arabidopsis*

## Abstract

Melatonin (*N*-acetyl-5-methoxytryptamine) plays important roles in regulating both biotic and abiotic stress tolerance, biological rhythms, plant growth and development. Sharing the same substrate (tryptophan) for the biosynthesis, melatonin and auxin also have similar effects in plant development. However, the specific function of melatonin in modulating plant root growth and the relationship between melatonin and auxin as well as underlying mechanisms are still unclear. In this study, we found high concentration of melatonin remarkably inhibited root growth in *Arabidopsis* by reducing root meristem size. Further studies showed that melatonin negatively regulated auxin biosynthesis, the expression of PINFORMED (PIN) proteins as well as auxin response in *Arabidopsis*. Moreover, the root growth of the triple mutant *pin1pin3pin7* was more tolerant than that of wild-type in response to melatonin treatment, suggesting the essential role of PIN1/3/7 in melatonin-mediated root growth. Combination treatment of melatonin and 5-Triiodobenzoic acid (TIBA) did not enhance melatonin-mediated reduction of root meristem size, indicating that polar auxin transport (PAT) may be necessary for the regulation of root meristem size by melatonin treatment. Taken together, this study indicates that melatonin regulates root growth in *Arabidopsis*, through auxin synthesis and polar auxin transport, at least partially.

## Introduction

Melatonin (*N*-acetyl-5-methoxytryptamine), a widely distributed endogenous bio-molecule in mammals, was first discovered in the bovine pineal gland in 1958 (Lerner et al., 1958). Melatonin regulates many important physiological processes in mammals, including sleep, body temperature regulation, circadian rhythms, mood, immune processes, etc. (Jan et al., 2009; Hardeland et al., 2012; Carrillo-Vico et al., 2013; Reiter et al., 2014).

The existence and discovery of melatonin in other species, especially in higher plants, indicates its extensive functions (Poeggeler et al., 1991; Dubbels et al., 1995; Hattori et al., 1995). Numerous studies have shown that melatonin is widely involved in regulating both the biotic and abiotic stress tolerance, biological rhythms, plant growth and development (seed germination, root architecture, shoot development, plant flowering, fruit ripening, and yield; Kang et al., 2010; Okazaki et al., 2010; Wang et al., 2012; Byeon et al., 2013, 2014b; Yin et al., 2013; Zhang et al., 2013, 2014; Zhao et al., 2013; Byeon and Back, 2014a; Shi and Chan, 2014; Weeda et al., 2014; Shi et al., 2015a,b,c,d,e, 2016).

In higher plants, melatonin is synthesized from tryptophan as substrate by four key enzymes [tryptophan decarboxylase (TDC), tryptamine 5-hydroxylase (T5H), serotonin *N*-acetyltransferase (SNAT), and *N*-acetylserotonin *O*-methyltransferase (ASMT)] (Kang et al., 2007a,b, 2011, 2013; Okazaki et al., 2009; Fujiwara et al., 2010; Byeon and Back, 2014b, 2015; Zuo et al., 2014; Arnao and Hernández-Ruiz, 2015). Recent studies found that *N*-acetylserotonin can also be synthesized via caffeic acid *O*-methyltransferase (COMT) in *Arabidopsis thaliana*, except ASMT (Byeon et al., 2014a; Lee et al., 2014). Interestingly, there are two different pathways for the synthesis of auxin (IAA) in plants, one is tryptophan-independent, and the other is tryptophan-dependent, sharing the same substrate with melatonin (Wang et al., 2015). Tryptophan-dependent pathway is dependent on precursor tryptophan, through tryptamine (TAM), indole-3-pyruvic acid (IPyA) and indole-3-acetaldoxime (IAOx) pathways (Benjamins and Scheres, 2008; Strader and Bartel, 2008; Chandler, 2009).

Melatonin also showed some similar functions as auxin in the higher plants, in addition to sharing the same substrate for their biosynthesis. Previous studies suggested that melatonin acts as a growth-stimulating molecule in *lupin* tissues and some monocots, including *canary grass*, *wheat*, *barely*, and *oat*; its IAA-like activity is 10–55% of that of auxin (Hernández-Ruiz et al., 2004, 2005). However, there are also reports indicating that melatonin regulates *Arabidopsis* root growth independent of auxin signaling (Pelagio-Flores et al., 2012; Koyama et al., 2013).

To date, the function of melatonin in regulating root growth and the underlying mechanisms are still unclear in higher plants. Moreover, the relationship between melatonin and auxin remains unknown. In the present work, different concentrations of melatonin were used to treat the wild-type (WT, Col-0) *Arabidopsis*. The results showed that melatonin significantly suppressed root growth by reducing the size of root meristem. Additionally, comprehensive analyses of auxin synthesis, PIN (PINFORMED) proteins and a auxin response marker line of *Arabidopsis* (DR5 promoter marker line) suggested that melatonin might regulate the root growth through auxin signaling, at least partially.

## Materials and Methods

### Plant Materials and Growth Conditions

The ecotype Columbia-0 (Col-0) of *Arabidopsis thaliana* was used as the WT plant in this study. Other plant materials are listed as follows: *pin1* (Salk_047613), *pin3* (CS9364), and *pin7* (CS9367) from the Arabidopsis Biological Resource Centre (ABRC), *pin3pin7* (Benková et al., 2003), *pin1pin3pin7* (Blilou et al., 2005), *PIN1::PIN1-GFP* (Benková et al., 2003), *PIN3::PIN3-GFP* (Blilou et al., 2005), *PIN7::PIN7-GFP* (Blilou et al., 2005), *DR5::GUS* (Sabatini et al., 1999), and *DR5::GFP* (Friml et al., 2003). *Arabidopsis* seeds were sterilized with 70% (v/v) ethanol for 1 min and 1% sodium hypochlorite for 16 min. After washing with distilled water for 3–5 times, seeds were sown on 1/2 Murashige and Skoog medium with 1% sucrose and 0.8% agar. The plates with seeds were placed at 4°C for 2 days to break dormancy prior to transfer to a culture room under dark/light cycles of 8 h/16 h at the temperature of 22°C. Plates were maintained in a vertical position for 3 days in the culture room before various treatments.

### Drug Treatments and Root Assay

As described above, 3-day-old *Arabidopsis* seedlings were transferred to 1/2 MS medium containing different concentrations of chemical components [melatonin, 2,3,5-Triiodobenzoic acid (TIBA) and IAA] for treatments. To limit the effect of solvent, the same volume of solvent including ethanol was used as a control. Thereafter, photos were taken by a digital camera, and the length of primary roots was determined by software Image J^1^ (version 1.47 g). For the root meristem size measurement, every five roots were cut and transferred onto a glass slide, and were treated with clearing solution (30 mL ddH_2_O, 53.3 g chloral hydrate and 10 mL glycerol) for 5 min before microscope analyses. Images were captured by Leica DM6000 differential interference contrast microscope. The zone between two white arrows in images include both the apical meristem and the transition zone (Verbelen et al., 2006; Baluska et al., 2010). Root meristem size was quantified as previously described (Liu et al., 2015; Yuan and Huang, 2016). Results presented are average values of more than 30 seedlings per treatment from three independent experiments. Statistical analysis was conducted in KaleidaGraph 4.03.

### GUS Staining

GUS staining was performed as described previously (Jefferson et al., 1987). Samples were cleared as mentioned above before observation. For *DR5::GUS* marker line, 2 h was enough for staining. The images of GUS staining were taken with a Leica DM6000 microscope equipped with Leica Application Suite software.

### Confocal Microscopy

*Arabidopsis* seedlings expressing *PIN1::PIN1-GFP*, *PIN3::PIN3-GFP*, *PIN7::PIN7-GFP*, and *DR5::GFP* were observed under Leica TCS SP8 laser scanning confocal microscope, with excitation of 488 nm argon laser, and emission wavelength range of 505–525 nm. The intensity of argon laser in laser configuration and intensity of laser line 488 in acquire section was set to 20 and 15%, respectively. Pinhole was set to 1.8 Airy units for all materials. To compare the fluorescent intensity of GFP in roots between control and samples treated, all optical sections were acquired under identical conditions. Quantification of the fluorescent intensity was performed by measuring the mean gray value using Image J software. Since PIN1 is mainly localized in the provasculature in roots, and that both PIN3 and PIN7 are expressed in provasculature and root cap. For the *PIN1::PIN1-GFP* roots, only the signals in the provasculature were quantified, while for *PIN3::PIN3-GFP* and *PIN7::PIN7-GFP* roots, signals both in the provasculature and root cap were quantified separately. And we did not distinguish signals at the plasma membrane from signals in the cytoplasm.

### Quantitative Real-Time PCR Analysis

Three-day-old *Arabidopsis* seedlings were transferred to new 1/2 MS medium and medium containing 600 μM melatonin. After another 7 days’ treatment, root tips (sections from root meristem to the tip) of control and samples were dissected under a dissecting microscope, and total RNA was isolated from root tips treated with TRIzol reagent (Invitrogen). For cDNA synthesis, 2 μg of total RNA from different samples was used for reverse transcription with RevertAid First Strand cDNA Synthesis Kit (Thermo Scientific) according to the manufacturer’s recommendations. To analyze the transcript levels of auxin-related genes in control and treated roots, quantitative real-time PCR was performed with Applied Biosystems 7500 (Foster City, CA, USA) in a 20-μL reaction volume containing SYBR Green dye (SYBR Premix Ex Taq, TAKARA). *PDF2* (protein phosphatase 2, AT1G13320) was chosen as an internal control (Czechowski et al., 2005). Relative expression levels were estimated using the 2^-ΔΔCt^ method (Livak and Schmittgen, 2001). All the primers used in the study are listed in Supplementary Table S1.

### Determination of Endogenous Melatonin and IAA Levels in *Arabidopsis* Roots

For the endogenous melatonin and IAA measurements, 3-days-old seedlings were transferred to new control 1/2 MS medium and medium containing IAA or melatonin for another 8 days. Endogenous melatonin in *Arabidopsis* root tips was extracted as previously described (Pape and Lüning, 2006). The levels of melatonin and IAA in root extracts were quantified using melatonin enzyme linked immunosorbent assay kit (EK-DSM; Buhlmann Laboratories AG, Schonenbuch, Switzerland) and Plant IAA enzyme-linked immunosorbent assay (EIASA) Kit (Jianglai Biotechnology, Shanghai, China), respectively, according to the instructions.

## Results

### Melatonin Suppressed the Primary Root Growth in *Arabidopsis* by Reduced Root Meristem

**FIGURE 1:**
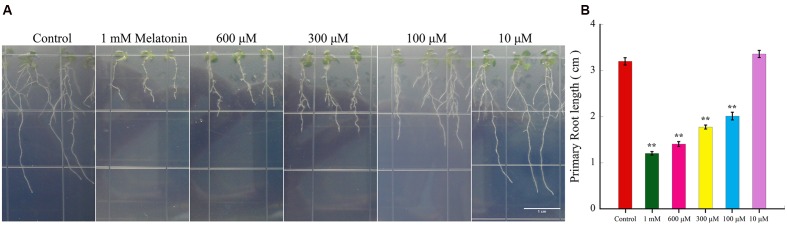
**Effect of high concentration of Melatonin on the length of *Arabidopsis* primary root**. After 3 days’ culture, seedlings were transferred to 1/2 MS medium with indicated concentrations of melatonin for other 6 days, and the primary root length were measured with software Image J. **(A)** Digital images of wild-type *Arabidopsis* seedlings treated with different concentrations of melatonin. Scale bar = 1 cm. **(B)** Primary root length of *Arabidopsis* growing on medium with control and increasing concentration of melatonin. More than 25 seedlings per experiment from three independent experiments were measured for statistic analysis. Values represent mean ± SD, ^∗∗^*P* < 0.01 by a Student’s *t*-test.

To investigate the effects of melatonin on primary root growth in *Arabidopsis*, 3-day-old WT (Col-0) seedlings were transferred to new 1/2 MS media with different concentrations of melatonin for another 6 days (**Figure 1**). By measuring and statistical analysis, we found that the primary root length was decreased after melatonin treatment, and the inhibition effect of melatonin exhibited dose-dependent (**Figure 1**). The result suggested that high concentration of melatonin could suppress the primary root growth in *Arabidopsis*.

**FIGURE 2:**
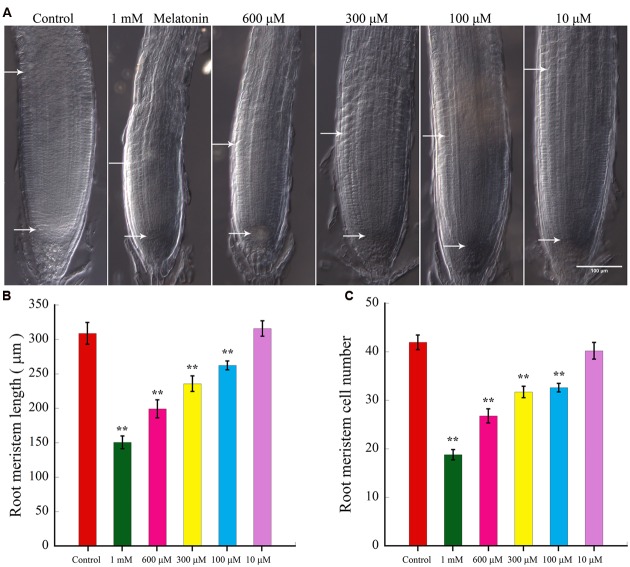
**Effects of Melatonin on the size of the *Arabidopsis* root meristem**. Three-day-old seedlings were kept growing under different concentrations of melatonin for other 6 days. More than 25 seedlings per experiment from three independent experiments were cleared for imaging. Values represent mean ± SD, ^∗∗^*P* < 0.01 by a Student’s *t*-test. **(A)** Images of *Arabidopsis* root tips treated with melatonin for 6 days were present. Scale bar = 100 μm. **(B)** Comparison of root meristem length of the *Arabidopsis* seedlings treated with different levels of melatonin. **(C)** Quantification of root meristem cell number of seedlings treated with different levels of melatonin. More than 25 seedlings per experiment from three independent experiments were measured for statistic analysis. Values represent mean ± SD, ^∗∗^*P* < 0.01 by a Student’s *t*-test.

In plants, postembryonic root growth is sustained by the root apical meristem (RAM), which consists of stem cell-like cells that are the precursors of all differentiated cell types (Laux and Mayer, 1998; Dinneny and Benfey, 2008). So we wonder if melatonin to reduce the primary root length by affecting root meristem. To test our hypothesis, 3-day-old seedlings were kept growing under different concentrations of melatonin for another 6 days, and we found that both the number of meristem cells and the length of meristem are significantly reduced with increased concentration of melatonin (**Figures 2B,C**), indicating that melatonin-mediated repression of primary root growth might be due to reduced root meristem.

Our data showed that 10 μM melatonin had no effect on primary root growth (**Figures 1** and **2**), and our previous work suggested that 10–50 μM melatonin had litter effect on endogenous melatonin content (Shi et al., 2015c). Therefore, we chose high concentration of melatonin for further analyses in this study.

### Melatonin Negatively Regulated Auxin Biosynthesis

**FIGURE 3:**
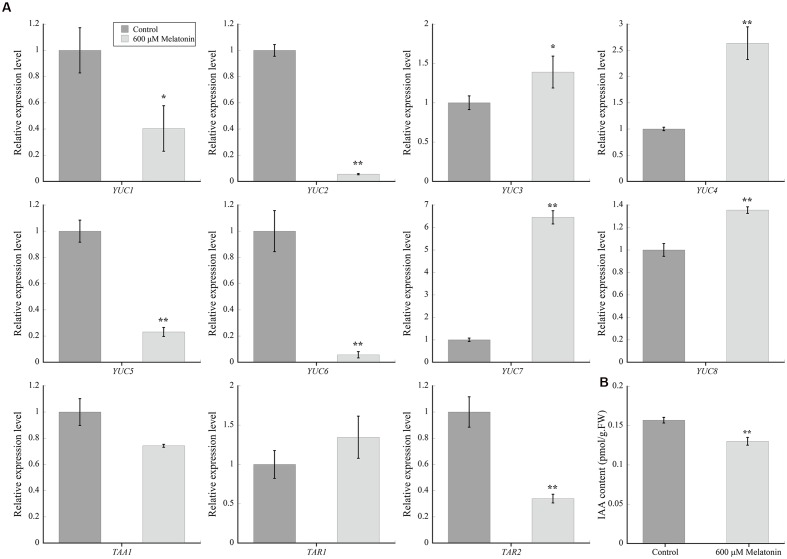
**The effects of melatonin on auxin synthesis and IAA content in *Arabidopsis* roots**. **(A)** qRT-PCR analysis of auxin synthesis related genes’ expression under control and 600 μM melatonin treatment. Relative fold changes of the expression of *YUC1*, *YUC2*, *YUC3*, *YUC4*, *YUC5*, *YUC6*, *YUC7*, *YUC8*, *TAA1*, *TAR1*, and *TAR2* were quantified by real-time PCR, and the expression levels of the indicated genes in control roots were set to 1. Values represent mean ± SD, ^∗^*P* < 0.05, and ^∗∗^*P* < 0.01 by a Student’s *t*-test. **(B)** IAA contents in the roots of seedlings grown on control medium or medium supplemented with 600 μM melatonin. Values represent mean ± SD, and ^∗∗^*P* < 0.01 indicate significant differences by a Student’s *t*-test.

Since defective auxin response can cause reduced meristem phenotype, the first question we wanted to known was whether melatonin actually affects auxin biosynthesis. YUCCA (YUC) proteins, TRYPTOPHAN AMINOTRANSFERASE OF ARABIDOPSIS (TAA) family, TAA RELATED 1 and 2 play important roles in auxin (IAA) biosynthesis during plant development (Cheng et al., 2006; Yamamoto et al., 2007; Stepanova et al., 2008; Tao et al., 2008), so we investigated the effects of melatonin on the transcript levels of these genes (**Figure 3A**). Quantitative real-time PCR showed that the transcript levels of *YUC1*, *YUC2*, *YUC5*, *YUC6*, and *TAR2* significantly decreased after 600 μM melatonin treatment. The transcript levels of *YUC3*, *YUC4*, *YUC7*, and *YUC8* increased after treatment, while the relative expression levels of *YUC3* and *YUC8* in roots with treatment were less than 1.5-fold in control. Indeed, the endogenous IAA content in melatonin-treated roots was significantly lower than that of control (**Figure 3B**).

### Melatonin Repressed Polar Auxin Transport in *Arabidopsis*

**FIGURE 4:**
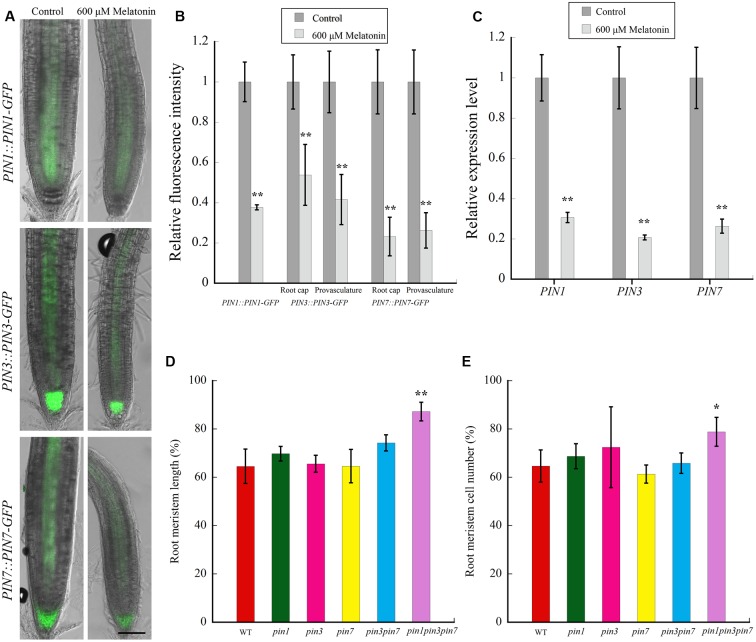
**The expression of auxin eﬄux components PINFORMEDS (PINs) were down-regulated after treatment of 600 μM melatonin**. **(A)** Effects of Melatonin on the abundance of PIN proteins in *Arabidopsis*. Three-day-old seedlings harboring indicated markers were transferred to control medium or medium with 600 μM melatonin for 6 days. Scale bar = 100 μm. **(B)** Comparison of GFP fluorescence intensity in plants treated without or with melatonin as in **(A)** by Image J. The fluorescence intensity levels of the control roots were set to 1. Values represent mean ± SD, ^∗∗^*P* < 0.01 by a Student’s *t*-test. **(C)** qRT-PCR analysis of *PIN1*, *PIN3*, and *PIN7* in *Arabidopsis* roots under 600 μM melatonin treatment. The expression levels of the indicated genes in control roots were set to 1. Values represent mean ± SD, ^∗∗^*P* < 0.01 by a Student’s *t*-test. **(D)** Quantification of relative root meristem length of various mutants treated without or with 600 μM melatonin for 6 days. Values represent mean ± SD, ^∗∗^*P* < 0.01 by a Student’s *t*-test. **(E)** Quantification of relative root meristem cell number of various mutants treated without or with 600 μM melatonin for 6 days. Values represent mean ± SD, ^∗^*P* < 0.05, and ^∗∗^*P* < 0.01 by a Student’s *t*-test.

PINFORMED proteins, especially PIN1, PIN3, and PIN7, directly participate in auxin transport in plant roots (Friml et al., 2003; Blilou et al., 2005), and play important roles in controlling the size of root meristem. In order to examine whether melatonin treatment affected the levels of these proteins in root, we measured the relative fluorescence intensity of GFP using the marker lines *PIN1::PIN1-GFP*, *PIN3::PIN3-GFP*, and *PIN7::PIN7-GFP*. As shown in **Figure 4**, signals of PIN1 in provasculture, and signals of PIN3 and PIN7 deriving from both root cap and provasculture region were decreased significantly after 600 μM treatment. The quantitative real-time PCR demonstrated that the relative transcript levels of *PIN1*, *PIN3*, and *PIN7* were also significantly reduced in melatonin-treated roots, suggesting that melatonin treatment repressed the expression of *PIN1*, *PIN3* and *PIN7*. To further confirm the involvement of PINs in melatonin-mediated root development, the meristem length and cell number of the roots of *PIN* mutants (including *pin1*, *pin3*, *pin7*, *pin3pin7*, and *pin1pin3pin7*) were also determined. Notably, we found that the root growth of triple mutant *pin1pin3pin7* was more tolerant to melatonin treatment than WT and other mutants (**Figures 4D,E**), indicating the essential role of PIN1/3/7 in melatonin-mediated repression of root meristem.

### Melatonin Repressed Auxin Response in *Arabidopsis* in an IAA Similar Manner

**FIGURE 5:**
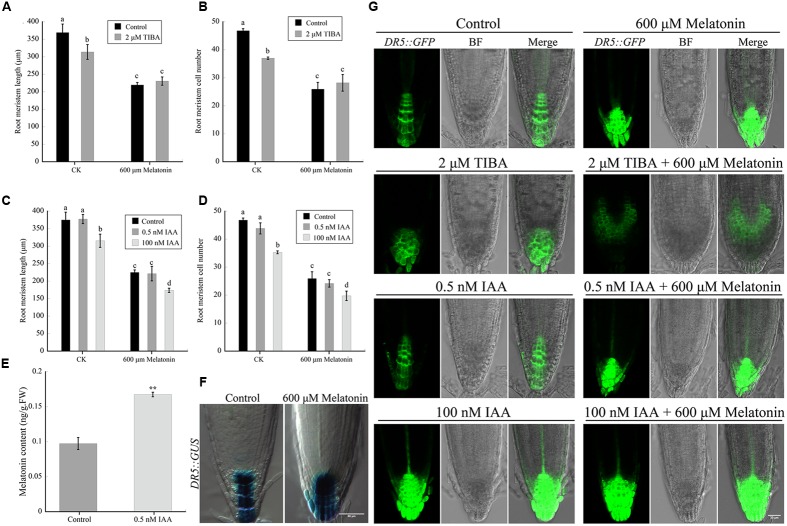
**Melatonin has similar effect as auxin on *Arabidopsis* root tip growth**. Three-day-old seedlings of wild-type (WT) and indicated markers were transferred to control medium or medium with various treatments. Values represent mean ± SD, ^∗^*P* < 0.05, and ^∗∗^*P* < 0.01 by a Student’s *t*-test. **(A)** Root meristem length of WT seedlings treated without or with 600 μM melatonin in the presence or absence of 2 μM TIBA for 8 days. **(B)** Root meristem cell number of WT seedlings treated without or with 600 μM melatonin in the presence or absence of 2 μM TIBA for 8 days. **(C)** Root meristem length of WT seedlings treated without or with 600 μM melatonin in the presence or absence of 0.5 or 100 M IAA for 8 days. **(D)** Root meristem cell number of WT seedlings treated without or with 600 μM melatonin in the presence or absence of 0.5 or 100 nM IAA for 8 days. More than 25 seedlings per experiment from three independent experiments were measured for statistic analysis. Values represent mean ± SD, different letters represent significant difference by a Student’s *t*-test. **(E)** Endogenous melatonin levels in the roots of seedlings grown on control medium or medium supplemented with 0.5 nM IAA. **(F)** Effect of melatonin on auxin response in root tips of *DR5::GUS* marker line seedlings. Scale bar = 50 μm. **(G)** Effect of melatonin, TIBA and IAA on auxin response in root tips of *DR5::GFP* marker line seedlings. Scale bar = 30 μm.

Endogenous auxin level is directly related to development of plant roots. To further dissect the underlying mechanism of melatonin during *Arabidopsis* root growth and the relationship between melatonin and auxin, exogenous IAA and auxin transport inhibitor (TIBA) were used to treat the seedlings. Firstly, 3-day-old seedlings were treated with melatonin containing medium in the presence or absence of 2 μM TIBA for 8 days. The root meristem length and cell number were measured. The results showed that both melatonin and TIBA treated roots reduced root meristem length and cell number, but the inhibition caused by melatonin was not intensified by the presence of TIBA (**Figures 5A,B**), indicating that polar auxin transport (PAT) might be necessary for the regulation of root meristem size by melatonin treatment.

Since melatonin-treated roots had lower IAA levels (**Figure 3B**), we wonder whether the root meristem size was reduced by melatonin through decreasing IAA content. If so, the reduction of root meristem size could be complemented, or partially complemented by exogenous IAA at a certain concentration. Two concentrations of IAA (0.5 and 100 nM) were used to treat the seedlings. We found that treatment of 0.5 nM IAA for 8 days alone did not affect the root meristem size, but 0.5 nM IAA and 600 μM melatonin co-treatment significantly reduced the root meristem size, similar to melatonin-treated roots alone (**Figures 5C,D**). Application of 100 nM IAA caused reduced root meristem size, as previous reported (Rahman et al., 2007; Strader et al., 2011), but the inhibition of 100 nM was less severe than that of 600 μM melatonin. To our surprise, 100 nM IAA and 600 μM melatonin co-treatment led to a more serious decrease in root meristem size than that of 600 μM melatonin (**Figures 5C,D**). In the meanwhile, we examined the content of endogenous melatonin in the roots of control and 0.5 nM IAA treated seedlings, and found that 0.5 nM IAA treatment resulted in increased level of melatonin (**Figure 5E**).

*DR5* promoter contains seven tandem repeat sequences of an auxin-responsive element, and it is widely used as a reporter for auxin signaling responses and auxin distribution in *Arabidopsis* (Ulmasov et al., 1997; Friml et al., 2003). After GUS staining, we found that the distribution of auxin was dramatically changed upon melatonin treatment, as it diffused into lateral root cap (LRC) cells from columella cells (**Figure 5F**). Similar results could be obtained by the observation of *DR5::GFP* line seedlings treated with melatonin for 5 days (**Figure 5G**) while 2 μM TIBA changed the expression pattern of *DR5::GFP* in root tips. In combination with melatonin, TIBA-induced auxin signals spread to LRC cells and adjacent meristem cells further (**Figure 5G**). Exogenous application of 100 nM IAA, but not that of 0.5 nM IAA, caused expansion of auxin signals to the lower part of LRC cells, similar to that of melatonin treatment. Interestingly, in association with melatonin, IAA-induced fluorescence signals spread to the whole distal tips of the roots below QC (**Figure 5G**), just like seedlings treated with higher concentrations of IAA as reported before (Ottenschläger et al., 2003), suggesting that melatonin aggravated the accumulation of auxin signals in the whole distal tips of roots, in an IAA similar manner.

## Discussion

As an important plant hormone, auxin plays vital roles in root cell division, differentiation, elongation, and the overall growth of roots (Benjamins and Scheres, 2008). In recent decades, more attention has been paid to the role of melatonin as a growth regulator of plants (Arnao and Hernández-Ruiz, 2015). Both of auxin and melatonin have been suggested to regulate similar growth processes. Our data showed 10 μM melatonin had no effect on primary root growth (**Figures 1** and **2**), and our previously work suggested that 10–50 μM melatonin had litter effect on endogenous melatonin content (Shi et al., 2015c). Based on previous studies and our preliminary experiments, 10–50 μM melatonin had litter effect on endogenous melatonin content (Shi et al., 2015c), and lower melatonin concentration also had litter effect on plant root development (Bajwa et al., 2014). Moreover, 100–600 μM melatonin were also widely used in other studies (Pelagio-Flores et al., 2012; Bajwa et al., 2014). In our study, we first tested the effect of melatonin on root growth of *Arabidopsis* and found that 100 μM melatonin had already shown an inhibitory effect on root growth, in accordance with previous reports (Chen et al., 2009). However, Bajwa et al. (2014) showed that 100, 200, and 400 μM melatonin treatment had no significant effects on plant root growth. The difference might be attributed to the big values of SD of their results and solvent effect. To limit the effect of solvent, the same volume of solvent including ethanol was used as a control in this study. Moreover, the average values of more than 30 seedlings per treatment from three independent experiments. The higher the concentration of melatonin, the more suppression of the root length (**Figure 1B**), indicating that melatonin inhibit root length in a dose-dependent manner. Although moderate auxin promotes root growth of plants, overproduction of auxin levels can cause a decay of root growth (Teale et al., 2005; Strader et al., 2011). After digging deeper into the effects of melatonin, we found high concentrations of melatonin reduced root meristem size, consistent with its effects on primary root length (**Figures 2B,C**).

The effects of auxin on root growth, is largely dependent on its biosynthesis and polar transport, which cause optimal auxin accumulation and distribution in the root apex during the whole developmental process (Blilou et al., 2005; Kim et al., 2007; Li et al., 2011). Quantitative real-time PCR showed that the expression levels of *YUC1*, *YUC2*, *YUC5*, *YUC6*, and *TAR2*, key genes of auxin biosynthesis, were significantly down-regulated after 600 μM melatonin treatment consistent with lower IAA content in 600 μM melatonin-treated roots (**Figure 3**). If we set the transcript level of *YUC1* in control material as 1, the relative expression level of *YUC2*, *YUC3*, *YUC4*, *YUC5*, *YUC6*, *YUC7*, *YUC8*, *TAA1*, *TAR1*, and *TAR2* was 28.7, 406.7, 4.6, 0.37, 151.9, 20.6, 170.3, 43.5, 41, and 693 separately. In melatonin-treated material, the relative transcript level of *YUC1* and other genes were 0.4, 1.6, 564.1, 11.9, 0.1, 1, 132.8, 230.8, 23.1, 29.6, and 234.3, respectively. After melatonin treatment, the total relative expression abundance was decreased significantly. The decrease in expression of *YUC1*, *YUC2*, *YUC5*, *YUC6*, and *TAR2*, together with the effects of melatonin on auxin transport may cause the decrease in IAA levels in roots, at least partially. As reported recently, application of 1-naphthaleneacetic acid (NAA) and 2,4-dichlorophenoxyacetic acid (2,4-D) results in a decay in the transcript levels of *YUC1*, *YUC2*, *YUC4*, *YUC6*, and *TAR2* in *Arabidopsis* seedlings (Suzuki et al., 2015). We noticed there is a difference in the endogenous IAA level between this study and previous results (Zhao et al., 2001; Lee et al., 2012), which could be resulted from two possible reasons. One reason could be the sample differences. In our work, only the root tips were harvested and used for analysis. Another possibility is that different methods were used. In this study, we used ELISA method. Based on the consistence between the transcript levels of auxin biosynthesis genes and IAA level, we concluded that melatonin negatively regulated auxin biosynthesis.

Polar auxin transport is essential for the distribution of auxin in *Arabidopsi*s root tips, and the auxin eﬄux machinery PIN proteins play important roles in controlling the size of root meristem (Friml et al., 2003; Blilou et al., 2005). Expression levels of PINs were always found down-regulated in the shortened root meristem after stresses (Liu et al., 2015; Yuan and Huang, 2016). Even in *PIN1::GFP* line roots exogenously treated with IAA, a significant decrease in GFP fluorescence was detected under high concentrations (5 and 10 μM; Omelyanchuk et al., 2016). Coincidently, our results demonstrated a decrease in both of the transcript levels and the protein expression levels of PIN1, PIN3, and PIN7 in melatonin-treated roots (**Figures 4A,C**, revised **Figure 4B**). Auxin transport inhibitor TIBA could also decrease the root meristem size and root growth (Blilou et al., 2005). Our study showed auxin transport inhibitor TIBA did not enhance melatonin-mediated reduction of root meristem size, indicating that PAT might be necessary for the regulation of root meristem size by melatonin treatment (**Figures 5A,B**). Moreover, the triple mutant *pin1pin3pin7* was more tolerant than WT in response to melatonin treatment, suggesting that PIN1/3/7-mediated PAT might contribute to melatonin-regulated root meristem.

Previous study showed application of low concentration of melatonin (0.1 μM), increased the endogenous levels of IAA in *Arabidopsis* roots (Chen et al., 2009), and our findings demonstrated that exogenous application of low concentration of IAA (0.5 nM), also raised the endogenous melatonin content in roots (**Figure 5E**). On the contrary, 100 nM IAA caused reduced root meristem size, as previously reported (Rahman et al., 2007; Strader et al., 2011). In this study, simultaneous presence of 100 nM IAA and 600 μM melatonin led to more serious decrease in root meristem size than that of 600 μM melatonin alone (**Figures 5C,D**).

The expression of *DR5* promoter marker line in root tips represents the responses and distribution pattern of auxin. Unlike the results showed by Pelagio and Koyama (Pelagio-Flores et al., 2012; Koyama et al., 2013), here we found that melatonin was able to change the expression pattern in both of *DR5::GUS* and *DR5::GFP* line roots (**Figure 5G**), just like the effects of IAA (Ottenschläger et al., 2003). In combination with 600 μM melatonin, 100 nM IAA caused a more expansion pattern of fluorescence signals in the whole root caps, including columella cells and lateral root cells, indicating that exogenous application of melatonin intensified the effect of IAA on the auxin responses in root tips. Considering altered auxin synthesis in root tip of treated seedlings, decreased signals of PIN1 in provasculature, reduced PIN3 and PIN7 signals in both root cap and provasculature, the DR5::GFP signal should not expand that strongly toward the lateral cap and tip cells after melatonin treatment. Then we also observed the expression and localization of PIN2 in the root cells, however, no significant difference was shown about the expression of PIN2 in cell membrane without and with 600 μM melatonin treatment (revised **Supplementary Figures S1A–C**). What’s surprised is that PIN2 signals in cytoplasm in melatonin-treated roots were obviously increased, although the signals in cytoplasm were much lower than that in cell membrane. This may, at least partially explain that why DR5 signals spread into the whole root cap after melatonin treatment. These results indicated that melatonin may have dual and complex effects on auxin transport. Besides PIN1/2/3/7, there may be other issues contribute to melatonin-mediated auxin signaling, which need to be further investigated.

**FIGURE 6:**
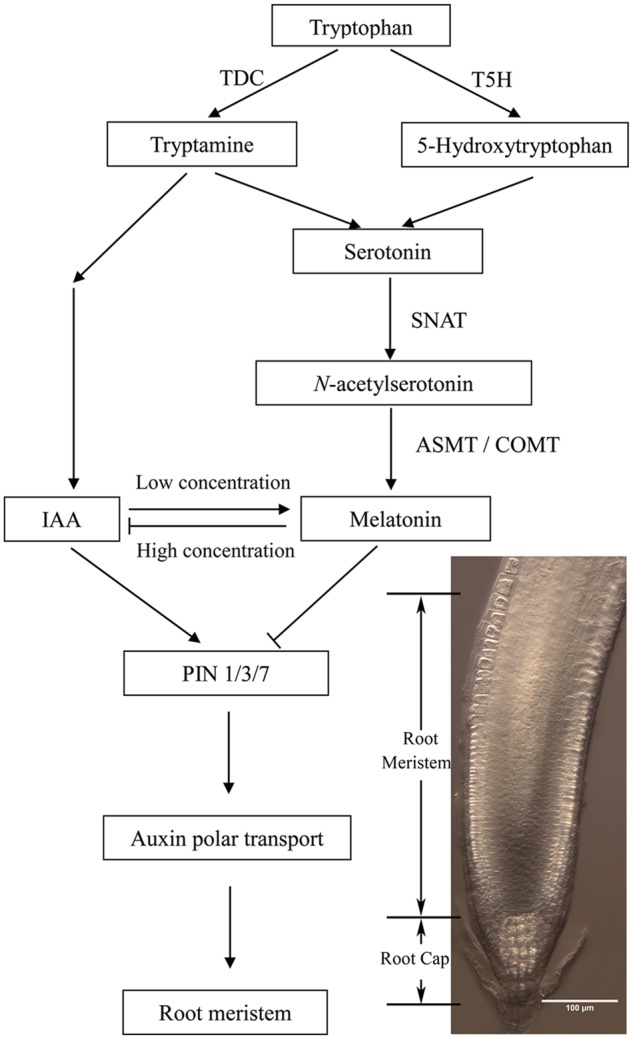
**The possible model showing the relationship between melatonin and auxin during plant root growth**.

Based on our results, we proposed a working model for the mechanisms by which melatonin regulates root meristem (**Figure 6**). Melatonin and auxin share the same substrate tryptophan during biosynthetic pathways, and exogenous application of IAA at low concentration increases melatonin production, while high concentration of melatonin decreases the level of IAA and PIN1, 3, 7 in *Arabidopsis* roots. Thus, melatonin regulates root meristem by repressing auxin synthesis and polar auxin transport in *Arabidopsis*. In summary, this study provides a direct link between melatonin and root growth, and indicates the novel involvement of auxin responses in melatonin-mediated root growth in *Arabidopsis*. We highlight the relationship between of melatonin and auxin in *Arabidopsis* root growth.

## Author Contributions

HS conceived and directed this study, revised the manuscript; QW designed and performed the experiments, analyzed the data, wrote and revised the manuscript; BA performed the experiments, analyzed the data and revised the manuscript; YW provided help in the melatonin and IAA content analysis; RR provided suggestions and revised the manuscript; HL designed the experiments and revised the manuscript; CH designed the experiments and revised the manuscript. All authors approved the manuscript and the version to be published.

## Conflict of Interest Statement

The authors declare that the research was conducted in the absence of any commercial or financial relationships that could be construed as a potential conflict of interest.

## Footnotes

1 http://rsbweb.nih.gov/ij/

## Supplementary Material

FIGURE S1 **The expression of PIN2 in cytoplasm of root cells was altered after 600 μM melatonin treatment**. Three-day-old seedlings harboring indicated markers were transferred to control medium or medium with 600 μM melatonin for 6 days. **(A)** Effects of Melatonin on the expression pattern of PIN2 in *Arabidopsis*. Scale bar = 50 μm. **(B)** Localization of PIN2 in root cells in control seedling and seedling treated with 600 μM melatonin. Scale bar = 5 μm. **(C)** Comparison of GFP fluorescence intensity in plants treated without or with melatonin as in **(A)** by Image J. The fluorescence intensity levels of the control roots were set to 1. Values represent mean ± SD, ^∗^*P* < 0.05, and ^∗∗^*P* < 0.01 by a Student’s *t*-test.

## Abbreviations

IAA: indolyl-3-acetic acid
TIBA: 5-Triiodobenzoic acid
