# Supplementary figures and images for: Melatonin Regulates Root Meristem by Repressing Auxin Synthesis and Polar Auxin Transport in Arabidopsis
Source: Front Plant Sci. 2016 Dec 15;7:1882. doi: 10.3389/fpls.2016.01882 (PMC5156734; doi:10.3389/fpls.2016.01882)

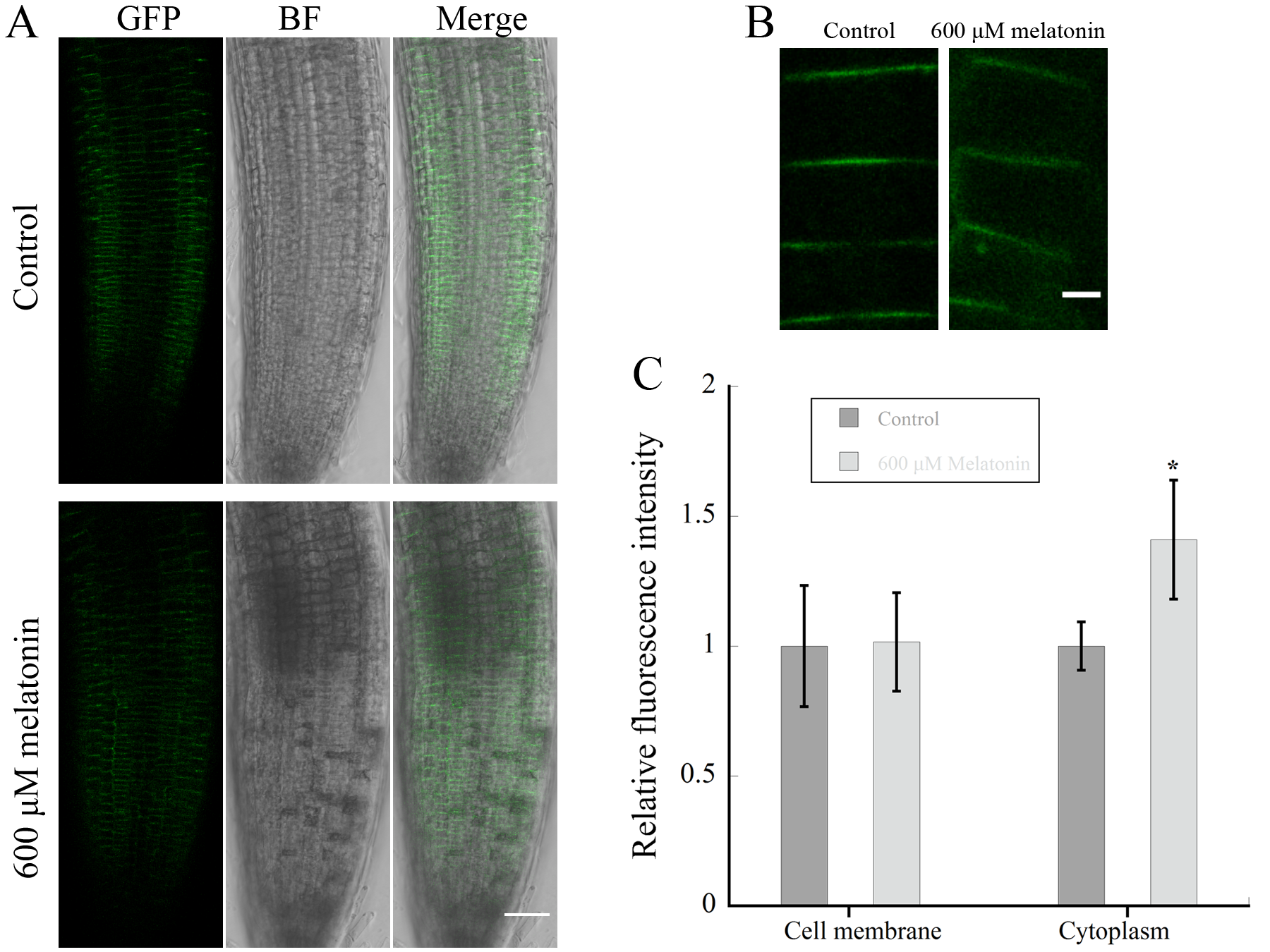

Supplement: FIGURE S1 — The expression of PIN2 in cytoplasm of root cells was altered after 600 μM melatonin treatment. Three-day-old seedlings harboring indicated markers were transferred to control medium or medium with 600 μM melatonin for 6 days. (A) Effects of Melatonin on the expression pattern of PIN2 in Arabidopsis. Scale bar = 50 μm. (B) Localization of PIN2 in root cells in control seedling and seedling treated with 600 μM melatonin. Scale bar = 5 μm. (C) Comparison of GFP fluorescence intensity in plants treated without or with melatonin as in (A) by Image J. The fluorescence intensity levels of the control roots were set to 1. Values represent mean ± SD, ∗P < 0.05, and ∗∗P < 0.01 by a Student’s t-test. [file Image_1.TIF]
