# Supplementary material for: Melatonin Regulates Root Meristem by Repressing Auxin Synthesis and Polar Auxin Transport in Arabidopsis
Source: Front Plant Sci. 2016 Dec 15;7:1882. doi: 10.3389/fpls.2016.01882 (PMC5156734; doi:10.3389/fpls.2016.01882)
Supplement: Supplementary file 2 [file Data_Sheet_1.DOC]

**Supplementary material**

**Supplemental Table S1.** List of the primers used in this study.

| **Primer** | **Sequence (5’ to 3’)** |
| --- | --- |
| *PDF2 F* | TAACGTGGCCAAAATGATGC |
| *PDF2 R* | GTTCTCCACAACCGCTTGGT |
| *PIN1 F* | GGAGACTTAAGTAGGAGCTCAGCA |
| *PIN1 R* | CCAAAAGAGGAAACACGAATG |
| *PIN3 F* | TCTTTGATTAGGTTCGGGTAACTC |
| *PIN3 R* | GCTCATGTGAAACTGGAACAAG |
| *PIN7 F* | CCAAGATTAGTGGAACGCAAC |
| *PIN7 R* | GAAAAGGGTTTTTGGATCCTC |
| *YUC1 F* | CAAAGAAAGGAGCAAAGTTTATGG |
| *YUC1 R* | CTGAAGCCAAGTAGGCACGTT |
| *YUC2 F* | GGTGACACGGATCGGTTAGGGT |
| *YUC2 R* | TGCCGAATAATGCATTACCCGT |
| *YUC3 F* | GATTTTGGCGGCGATGTT |
| *YUC3 R* | TTTGCTCCGTGATTGTAG |
| *YUC4 F* | AAAACTCCCGTTCTTGATGTCG |
| *YUC4 R* | TCTTTCCCATTCAGAAACTTTGC |
| *YUC5 F* | GGGTTAACGGTCCTGTAATCGT |
| *YUC5 R* | TCTGCTCTCTCCAATACCACAAAG |
| *YUC6 F* | CTCGTTGTCAGAGACGCTGT |
| *YUC6 R* | AACCAAAAGGAAACGGTCAA |
| *YUC7 F* | TGATTCTTGCCACTGGTTACAGA |
| *YUC7 R* | CCTTCCCTTTCCACCCTTTT |
| *YUC8 F* | TGTATGCGGTTGGGTTTACG |
| *YUC8 R* | CAGAGCCTATGTCTTGTGCGAT |
| *TAA1 F* | CCCCACTACACTCCCATCACTC |
| *TAA1 R* | TCACCAATGCCCACCCAATAC |
| *TAR1 F* | GCAACTTCTTTGGCAAAACCC |
| *TAR1 R* | ATCGGTCTCCTCCTCTCGTCA |
| *TAR2 F* | TTGGGGTTTCAAAGGACTCAC |
| *TAR2 R* | TTTGCTGCTTGTTTCAATAGTTTC |
